# Multiparametric MRI enables for differentiation of different degrees of malignancy in two murine models of breast cancer

**DOI:** 10.3389/fonc.2022.1000036

**Published:** 2022-11-02

**Authors:** Mirjam Gerwing, Emily Hoffmann, Katharina Kronenberg, Uwe Hansen, Max Masthoff, Anne Helfen, Christiane Geyer, Lydia Wachsmuth, Carsten Höltke, Bastian Maus, Verena Hoerr, Tobias Krähling, Lena Hiddeßen, Walter Heindel, Uwe Karst, Melanie A. Kimm, Regina Schinner, Michel Eisenblätter, Cornelius Faber, Moritz Wildgruber

**Affiliations:** ^1^ Clinic of Radiology, University of Münster, Münster, Germany; ^2^ Translational Research Imaging Center, University of Münster, Münster, Germany; ^3^ Institute of Inorganic and Analytical Chemistry, University of Münster, Münster, Germany; ^4^ Institute for Musculoskeletal Medicine, University of Münster, Münster, Germany; ^5^ Heart Center Bonn, Department of Internal Medicine II, University of Bonn, Bonn, Germany; ^6^ Department of Radiology, University Hospital, Ludwig-Maximilian University, Munich, Germany; ^7^ Department of Diagnostic and Interventional Radiology, University of Freiburg, Freiburg, Germany

**Keywords:** oncologic imaging, tumor heterogeneity, tumor vasculature, MRI, LA-ICP-MS

## Abstract

**Objective:**

The objective of this study was to non-invasively differentiate the degree of malignancy in two murine breast cancer models based on identification of distinct tissue characteristics in a metastatic and non-metastatic tumor model using a multiparametric Magnetic Resonance Imaging (MRI) approach.

**Methods:**

The highly metastatic 4T1 breast cancer model was compared to the non-metastatic 67NR model. Imaging was conducted on a 9.4 T small animal MRI. The protocol was used to characterize tumors regarding their structural composition, including heterogeneity, intratumoral edema and hemorrhage, as well as endothelial permeability using apparent diffusion coefficient (ADC), T1/T2 mapping and dynamic contrast-enhanced (DCE) imaging. Mice were assessed on either day three, six or nine, with an i.v. injection of the albumin-binding contrast agent gadofosveset. Ex vivo validation of the results was performed with laser ablation-inductively coupled plasma-mass spectrometry (LA-ICP-MS), histology, immunhistochemistry and electron microscopy.

**Results:**

Significant differences in tumor composition were observed over time and between 4T1 and 67NR tumors. 4T1 tumors showed distorted blood vessels with a thin endothelial layer, resulting in a slower increase in signal intensity after injection of the contrast agent. Higher permeability was further reflected in higher K_trans_ values, with consecutive retention of gadolinium in the tumor interstitium visible in MRI. 67NR tumors exhibited blood vessels with a thicker and more intact endothelial layer, resulting in higher peak enhancement, as well as higher maximum slope and area under the curve, but also a visible wash-out of the contrast agent and thus lower K_trans_ values. A decreasing accumulation of gadolinium during tumor progression was also visible in both models in LA-ICP-MS. Tissue composition of 4T1 tumors was more heterogeneous, with intratumoral hemorrhage and necrosis and corresponding higher T1 and T2 relaxation times, while 67NR tumors mainly consisted of densely packed tumor cells. Histogram analysis of ADC showed higher values of mean ADC, histogram kurtosis, range and the 90^th^ percentile (p90), as markers for the heterogenous structural composition of 4T1 tumors. Principal component analysis (PCA) discriminated well between the two tumor models.

**Conclusions:**

Multiparametric MRI as presented in this study enables for the estimation of malignant potential in the two studied tumor models *via* the assessment of certain tumor features over time.

## 1 Introduction

In the past decade, a variety of specific features that tumors acquire during progression and metastasis was identified and is summarized in the Hallmarks of Cancer concept (1). Tumor heterogeneity defined as the coexistence of different biological, morphological, phenotypic and genotypic profiles is an important feature. It occurs between tumors (intertumorally), within different tumor regions (intratumorally), between primary cancer and metastases (spatial heterogeneity) or during the course of disease progression (temporal heterogeneity) (2, 3). A high degree of heterogeneity is a key feature of malignancy and adversely affects patient’s prognosis, therapy response and clinical outcome due to consecutive tumor progression, metastasis formation and therapy resistance (4). It is further one of the main causes of failure in clinical cancer therapy (2), as specific activating gene mutations or expression levels of certain therapeutic targets are not present on all cancer cells alike (5).

Therefore, it is essential to monitor the evolution of disease under therapy as well as upon relapse, to appreciate cancer plasticity, mutational spectrum and clonal evolution during disease progression (4). Evaluation of tumor features with classical laboratory analyses such as histology after biopsy is limited, as repeated biopsies cannot be routinely performed in a frequent manner and tissue biopsies do not capture intratumoral heterogeneity due to a high sampling bias. It is thus crucial to assess the heterogeneity of the primary tumor and possible metastatic sites to characterize the tumor and decide on the appropriate therapy (6–8). Dedicated non-invasive techniques to evaluate these characteristics and their changes both over time during diseases progression and therapy are therefore needed – for identification of suitable patients for a specific anti-cancer therapy and detection of non-responders early after therapy initiation. A number of different imaging approaches have been introduced to meet this challenge (9), spanning the entire available range of imaging modalities, from ultrasound to PET with specific tracers. However, only multiparametric MRI is able to assess different tumoral features as a one-stop-shop, non-invasive, radiation-free examination (10). The obtained MRI results were referenced to several ex vivo analyses in this study, namely quantification of the extravasated gadolinium with laser-ablation inductively-coupled mass spectrometry (LA-ICP-MS), and detailed analysis of tumor composition with histology and immunohistochemistry. It has furthermore been shown, that multiparametric MRI co-registered with histology can define physiologically distinct tumor habitats within breast cancer models (10).

We hypothesize that a multiparametric MRI approach is able to capture certain distinct features of tumor heterogeneity, over time as well between the high malignant 4T1 and low malignant 67NR tumor model, including vascular permeability, intratumoral edema and hemorrhage as well as tumor cellularity. This can be used to assess the primary tumor, as well as metastases and their changes over time and might help to differentiate highly malignant, metastasizing tumors from ones with lower degree of malignancy.

## 2 Materials and methods

### 2.1 Mouse models

Female BALB/c mice were purchased from Charles River Laboratories (Wilmington, MA, U.S.A.) and used at eight to twelve weeks of age. All animal experiments in this study were carried out in accordance with local animal welfare guidelines and have been approved by the responsible state agency for nature, environment and consumer protection of North Rhine-Westfalia, Germany, (LANUV; approval ID 81-02.04.2018.A010). Mice were kept under a 12h light-dark cycle and provided with food and water ad libitum.

Syngeneic murine mammary carcinoma models with a highly metastatic and non-metastatic malignant variant were used. Highly malignant 4T1 tumors invade in the surrounding tissue, shed cells, and metastasize to regional lymph nodes as well as distant organs, primarily the lung, liver and bones (11, 12). 67NR tumors share the same genetic background, but do not shed cells or develop metastases (12). This model is an established syngeneic model system of graded malignancy (13).

### 2.2 Cell culture

Murine cell lines 4T1 and 67NR were cultured in Dulbecco’s modified Eagle Medium (DMEM; Thermo Fisher Scientific, Waltham, MA, U.S.A.), supplemented with 10% fetal calf serum. Mycoplasm contamination was excluded with PCR using a VenorGeM-OneStep test kit (Minerva Biolabs, Berlin, Germany) twice a week.

### 2.3 Tumor implantation

Cells with a confluency of approximately 80% were harvested and counted using a TECAN Reader (Tecan Group AG, Männedorf, Germany). 1 x 10^6^ 4T1 or 67NR cells were resuspended in 25 µl cell culture medium and injected orthotopically in the left mammary fat pad of the mice. During implantation, mice were kept under isoflurane anesthesia with 1.5% isoflurane and 2 l/minute of oxygen.

### 2.4 MRI

MR imaging was performed on a 9.4 T Biospec system (Bruker Biospin, Ettlingen, Germany), with a 1H quadrature volume resonator and a 10 mm surface coil, using the ParaVision 6.0.1. software for acquisition. Mice were anesthetized with 1.5% isoflurane in 1 l/minute of oxygen and compressed air (20:80) under continuous respiratory and temperature monitoring (SA Instruments, Stony Brook, NY, USA). They were placed in supine position and the tumor was covered in alginate (Johannes Weithas, Lütjenburg, Germany) to reduce susceptibility artefacts. Net examination time was 56:15 min for the entire protocol. For anatomical information, a T2 weighted rapid acquisition relaxation enhanced (RARE) sequence with 2500 ms TR, 55 ms TE, 2 averages, 1 repetition, 20 x 20 mm FOV, 1 mm slice thickness and an acquisition time of 1 min 45 s was acquired. T2 weighted imaging furthermore enabled to assess the tumor size three-dimensionally and evaluate intratumoral changes caused by different distribution of intratumoral fluids. Therein, the slice with the greatest mean tumor diameter served as reference for the following single-slice sequences: Firstly, a diffusion-weighted echo planar imaging (DW-EPI) sequence with 3000 ms TR, 21.5 ms TE, 8 averages, 1 repetition, 1 mm slice thickness, 18 x 18 mm field of view (FOV) and the b-values 20, 100, 200, 300, 400, 600, 800, 1000, 1200, 1500, 1800 and 2200 s/mm^2^, gradient width 3 ms, gradient distance 8.9 ms, with 5 min 12 s acquisition time. The mean apparent diffusion coefficient (ADC) was calculated by the Bruker software. Afterwards, a histogram analysis of ADC values of the tumor was performed using GrahPadPrism (version 9.2.0, GraphPad Software Inc., San Diego, CA, USA). The analyzed parameters included kurtosis (sharpness of the peak of the frequency-distribution curve), range (difference between maximum and minimum value) and 90^th^ percentile (the values above the 90^th^ percentile, p90). The 90^th^ percentile yielded the most distinct results and was thus chosen for this study. Regions of interest (ROI) were drawn around the entire tumor by one investigator (E.H.), on the obtained single slice image, to assess all measurable intratumoral changes. T2 weighted images were used as guidance. Combined T1 and T2 mapping was acquired before and after injection of the contrast agent, with a RARE sequence (TR 7500, 5000, 3000, 1500, 800, 400, 311, 123 ms, TE 90, 70, 50, 30, 10 ms, 1 average, 1 repetition, RARE spin echo factor 2, 1 mm slice thickness, 18 x 15 mm FOV and acquisition time 14 min 39 s). T1 and T2 relaxation times were calculated with the image sequence analysis tool within ParaVision. T1 maps were calculated from image series with different TR by voxel-wise fitting of the signal intensity with a mono-exponential function SI(TR) = A + C * (1 - exp^(-TR/T1)), with SI = voxel signal intensity; A = absolute bias/offset; C = proton density. T2 maps were calculated from image series with different TE by voxel-wise fitting of the signal intensity with a mono-exponential function SI(TE) = A + C * exp^(-TE/T2). Relaxation times were then retrieved from these maps based on ROI copied from the ADC maps (14). Histogram analysis (entropy of T1 maps, interquartile range (IQR) of T2 maps and entropy and skewness of T1 maps after injection of gadovosfeset) was performed using 3D Slicer (version 4.11.2021, USA) (15).

For dynamic contrast-enhanced (DCE) imaging, the albumin-binding contrast agent gadofosveset trisodium (Lantheus Medical Imaging, North Billerica, MA, U.S.A.) was injected *via* a tail vein catheter (Klinika Medical GmbH, Usingen, Germany) one minute after start of the DCE fast low-angle shot (FLASH) scan (TR 24.6 ms, TE 1.5 ms, 1 average, 610 repetitions, 18 × 15 mm FOV, acquisition time 20 min) with a perfusion pump (World Precision Instruments, Sarasota, FL, U.S.A.), in a concentration of 0.6 mmol/kg and a rate of 240 µl/min. Dynamic assessment of contrast enhancement enables to calculate different perfusion parameters, K_trans_, area under the curve (AUC) and maximum slope, which emphasize different characteristics of the enhancement over time. Analysis of the maps was performed with Image J (16) and post-processed with GraphPadPrism (version 9.2.0, GraphPad Software Inc., San Diego, U.S.A.). Calculation of perfusion parameters were performed with an in-house developed software based on the PkModeling extension for 3D Slicer (https://github.com/millerjv/PkModelings) with an extended TOFTS model, using a population-based arterial input function (17) and extrapolation of the longitudinal relaxivities of the applied contrast agent (18). After the MRI scan, mice were sacrificed and the tumor removed. For each tumor model and time point, at least n=8 mice were scanned and analyzed.

### 2.5 Laser-ablation-inductively coupled plasma-mass spectrometry

A possibility to assess distribution of gadolinium with a high spatial resolution of 15 µm is LA-ICP-MS, which was conducted after the MRI scans. After removal, tumors were immediately frozen in liquid nitrogen and stored at -80°C until further use. For LA-ICP-MS analysis, matrix-matched standards based on gelatin were prepared for external calibration (19). For this purpose, stock solutions with 1000 mg/L gadolinium (GdCl_3_·6 H_2_O) and 1000 mg/L Fe (FeSO_4_·7 H_2_O) were prepared and diluted in doubly distilled water (ddH_2_O) to achieve concentrations between 0 and 600 mg/L Gd or Fe, respectively. For the standards, 100 mg gelatin were spiked with 900 µL of the differently concentrated solutions of Gd and Fe, heated up to 60°C and mixed until homogenous. For further analysis with LA-ICP-MS, 10 µm thin sections of the standards were prepared using a cryomicrotome (CryoStar™ NX70, Thermo Fisher, Bremen, Germany or CM1850, Leica Biosystems, Wetzlar, Germany). To validate the concentrations in the gelatin standards, bulk analysis was performed after acidic digestion by means of ICP-MS (7700x ICP-MS, Agilent Technologies, Santa Clara, CA, U.S.A.). For each standard, 50 mg were digested with 1 ml of HNO_3_ (conc.) and Rh was added as internal standard and filled up to 50 ml with ddH_2_O to a final Rh concentration of 1 µg/L. Quantification for bulk analysis was achieved using an external calibration of a diluted Gd ICP-MS standard solution in a concentration range between 0 and 30 µg/L.

For quantitative bioimaging, 10 µm thin sections of the tumors were prepared with the same cryomicrotome as used for the gelatin standards. For analysis of all sections, a LSX 213 G2+ (CETAC Technologies, Omaha, NE, U.S.A.) laser ablation system equipped with a two volume HelEx II ablation cell was used. The laser ablation system was connected *via* Tygon tubing directly to the 7700x ICP-MS (Agilent Technologies, Santa Clara, CA, U.S.A.). For external calibration, ten lines of each standard were ablated, and the averaged intensities of the validated element concentrations were analyzed with a weighted linear regression (20). Ablation of tumor slices was performed by a line-by-line scan. The standards and samples were ablated using a laser spot size of 15 µm, a scan speed of 30 µm/s, a repetition rate of 20 Hz and a He flow of 800 mL/min as transport gas. Laser energy was adjusted for quantitative ablation. Experiments were conducted in a collision gas mode with He as collision gas and an integration time of 60 ms for the isotopes ^31^P, ^13^C and ^66^Zn, and 100 ms for ^56^Fe, ^57^Fe and ^158^Gd. The LOD and LOQ, calculated according to 3σ- and 10σ-criteria, were in a range between 0.024 - 0.21 µg/g and 0.081 - 0.69 µg/g for Gd and 1.1 - 15 µg/g and 3.7 - 51 µg/g for Fe, respectively. Quantification and visualization of the analyzed elements were carried out with an in-house developed software (ImaJar 3.64, written by Robin Schmid).

For both tumor models and all time points, three samples (triplicates) with one section each were analyzed by LA-ICP-MS and the averaged Gd concentration for every section was calculated. Based on these values, the mean of the triplicates and the sample standard deviation (STD) were determined and displayed as mean ± STD.

### 2.6 Electron microscopy

Small (approximately 2 mm) pieces of tumor samples were fixed overnight at 4°C in 2% (v/v) formaldehyde and 2.5% (v/v) glutaraldehyde in 100 mM cacodylate buffer, pH 7.4. After washing in PBS, samples were postfixed in 0.5% (v/v) osmiumtetroxide and 1% (w/v) potassium hexacyanoferrate (III) in 0.1 M cacodylate buffer for 2h at 4°C and subsequently washed with distilled water. After dehydration in an ascending ethanol series from 30 to 100% ethanol, samples were incubated in propylenoxide twice for 15 min each.

Subsequently, small tissue pieces were embedded in Epon (Sigma Aldrich, St. Louis, MO, U.S.A.) using flat embedding molds. Ultrathin sections (80 nm) were cut in an ultramicrotome, collected on copper grids and negatively stained with 2% uranyl acetate for 15 min. Electron micrographs were taken at Philipps EM-410 electron microscope (Philipps, Amsterdam, The Netherlands) using imaging plates (Ditabis, Pforzheim, Germany).

### 2.7 Histology and immunohistochemistry

For subsequent histological analysis, tumors were paraffin-embedded according to standard protocols and 5 µm slices prepared using a rotary microtome (Leica Camera AG, Wetzlar, Germany). Standard hematoxylin and eosin staining was performed as published previously (21). Staining of CD31 and Ki67 staining was done as previously described (22). In brief, both stainings were started with dewaxing and rehydration, followed by incubation in unmasking solution (Vector Laboratories, Newark, CA, U.S.A.) for 10 min in a pressure cooker and incubation in 3% H_2_O_2_ for 10 minutes. For CD31 staining, the protocol of a Vectastain kit was followed (PL-6101, Vector Laboratories, Newark, CA, U.S.A.). Primary antibody CD31 (ab28364, Abcam, Cambridge, U.K.) was used at a dilution of 1:200 for 120 minutes at room temperature followed by HRP/DAB detection. For Ki67 fluorescence staining, primary antibody Ki67 (ab15580, Abcam, Cambridge, U.K.) was used at a 1:100 dilution overnight at 4° C, followed by secondary antibody anti-rabbit Alexa 647 (Jackson 111-605-144, Jackson ImmunoResearch, Baltimore Pike, PA, U.S.A.), diluted at 1:200 in DAPI-solution (#46190, Thermo Fisher Scientific Inc., Waltham, MA, U.S.A.) and incubated for 45 min at room temperature.

### 2.8 Principal component analysis

Principal component analysis enables to visually assess similarities and differences between samples and determine whether samples can be grouped. It is especially helpful in analyses that include a larger number of variables and was used in this study to include all MRI results in a single analysis in order to determine if the two tumor models can be clearly distinguished based on the MRI data (23). ADC_mean_, ADC histogram kurtosis, ADC histogram range, ADC histogram p90, ΔT1 relaxation time, K_trans_, AUC, slope_max_, T1 relaxation time and T2 relaxation time were the included variables. PCA was performed by calculating ten principal components using SPSS (version 28.0, IBM, Armonk, New York, USA) as well as GraphPadPrism for visualization.

### 2.9 Statistics

Data are presented as mean ± standard deviation (STD). The Shapiro-Wilk-Test was used to test the values for normal distribution, if the p-value was <0.05, the values were considered not normally distributed and thus, Mann-Whitney-U was used for the comparison between the two tumor models and Kruskal-Wallis for changes over time within one tumor model (days 3, 6 and 9). If the p-value of the Shapiro-Wilk-Test was >0.05, a normal distribution was assumed, and a t-test was used to compare the values of the 4T1 and 67NR tumor model, while ANOVA was used to compare the values over time within one model (3 vs. 6 and 9 days). Statistics were calculated using SAS Version 9.4 for Windows (SAS Institute Inc., Cary, NC, U.S.A). Correlation analysis of the delta T1 relaxation times and the Gd concentration acquired by means of LA-ICP-MS was performed using Pearsons correlation method with GraphPadPrism (version 9.2.0, GraphPad Software Inc., San Diego, CA, USA).

P-values below 0.05 were considered significant (* p < 0.05, ** p < 0.01, *** p < 0.001, **** p < 0.0001).

## 3 Results

### 3.1 Volumetry and anatomical T2 weighted images

**Figure 1:**
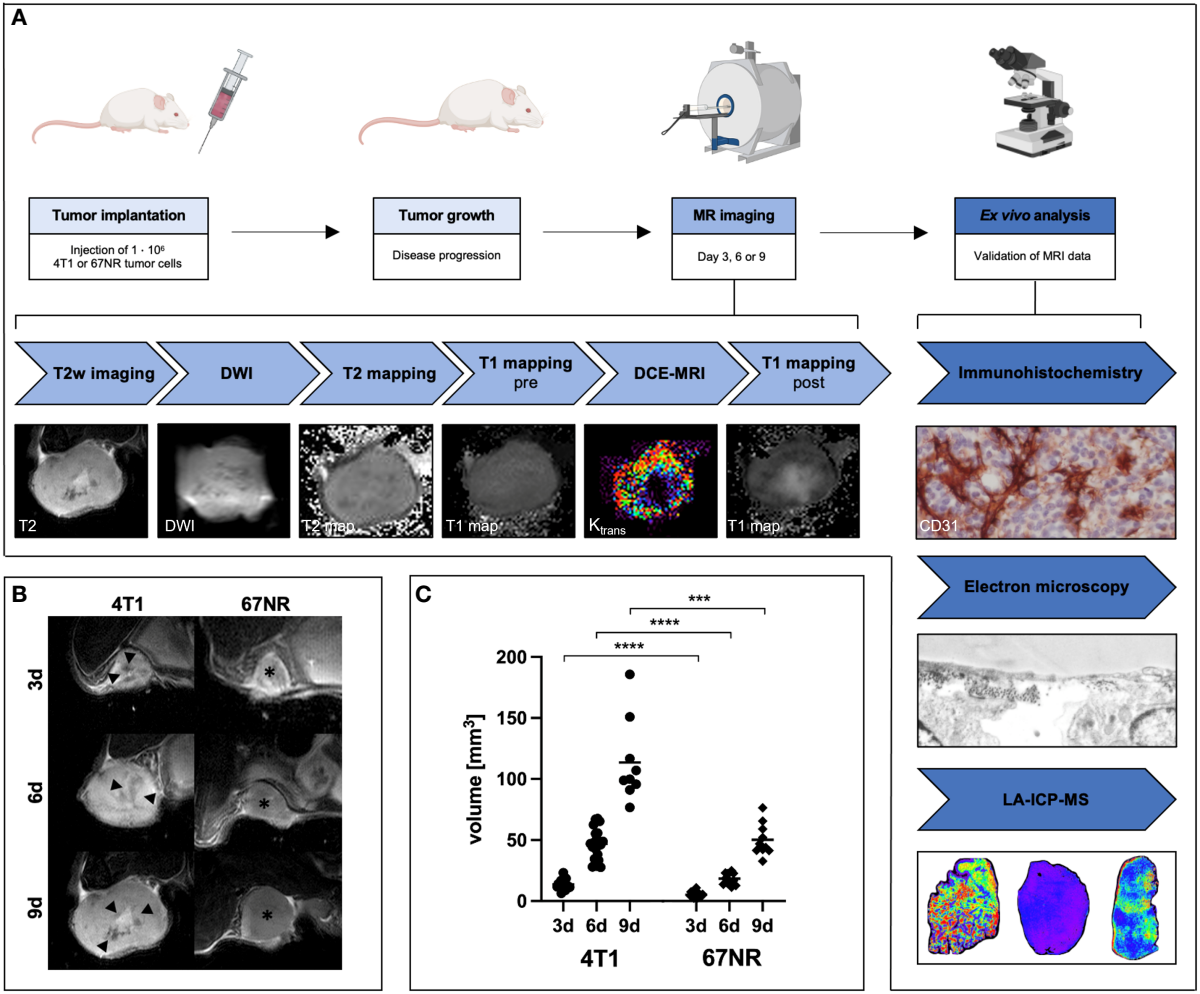
Overview over experimental setup and assessment of T2 weighted MRI images over time. BALB/c mice were implanted with 4T1 or 67NR tumor cells in the left mammary fat pad and let grow for three, six or nine days. After the multiparametric MRI scans, mice were sacrificed, and the tumors removed for *ex vivo* analyses **(A)**. T2 weighted images **(B)** of exemplary 4T1 tumors (left side) and 67NR tumors (right side), and volumetry of the tumors **(C)** show an increasing discrepancy between the two tumor models during progression. 4T1 tumors measured double the size of 67NR tumors on day nine. Note the already visible small necrotic/hemorrhagic areas in the 4T1 tumors (arrowheads) in comparison to the homogenous 67NR tumors (asterisks). ***p < 0.001, ****p < 0.0001. Partially created with BioRender.com **(A)**.

The single values for each time point and sequence (mean values and standard deviation of the tumor ROI), including the p-values, can be found in **Supplementary Table 1**. These have not been included in the manuscript for better readability. Tumors were detectable on day three for both cell lines, with an exponential growth of the 4T1 tumors to day six and nine, while 67NR tumors remained smaller. On day nine, 67NR tumors measured approximately half the size of 4T1 tumors. Tumor size was significantly different between the tumor models at all time points, as were the changes over time. T2w images visualized homogenous tumors for the 67NR model, while 4T1 tumors revealed heterogenous intratumoral fluid distribution, increasing over time with areas of hypointense signal changes, corresponding to necrosis and hemorrhage (**Figure 1**). **Figure 1** also includes a detailed overview over the experimental setup with exemplary MR images and ex vivo analyses.

### 3.2 Assessment of the structural composition with diffusion-weighted imaging

**Figure 2:**
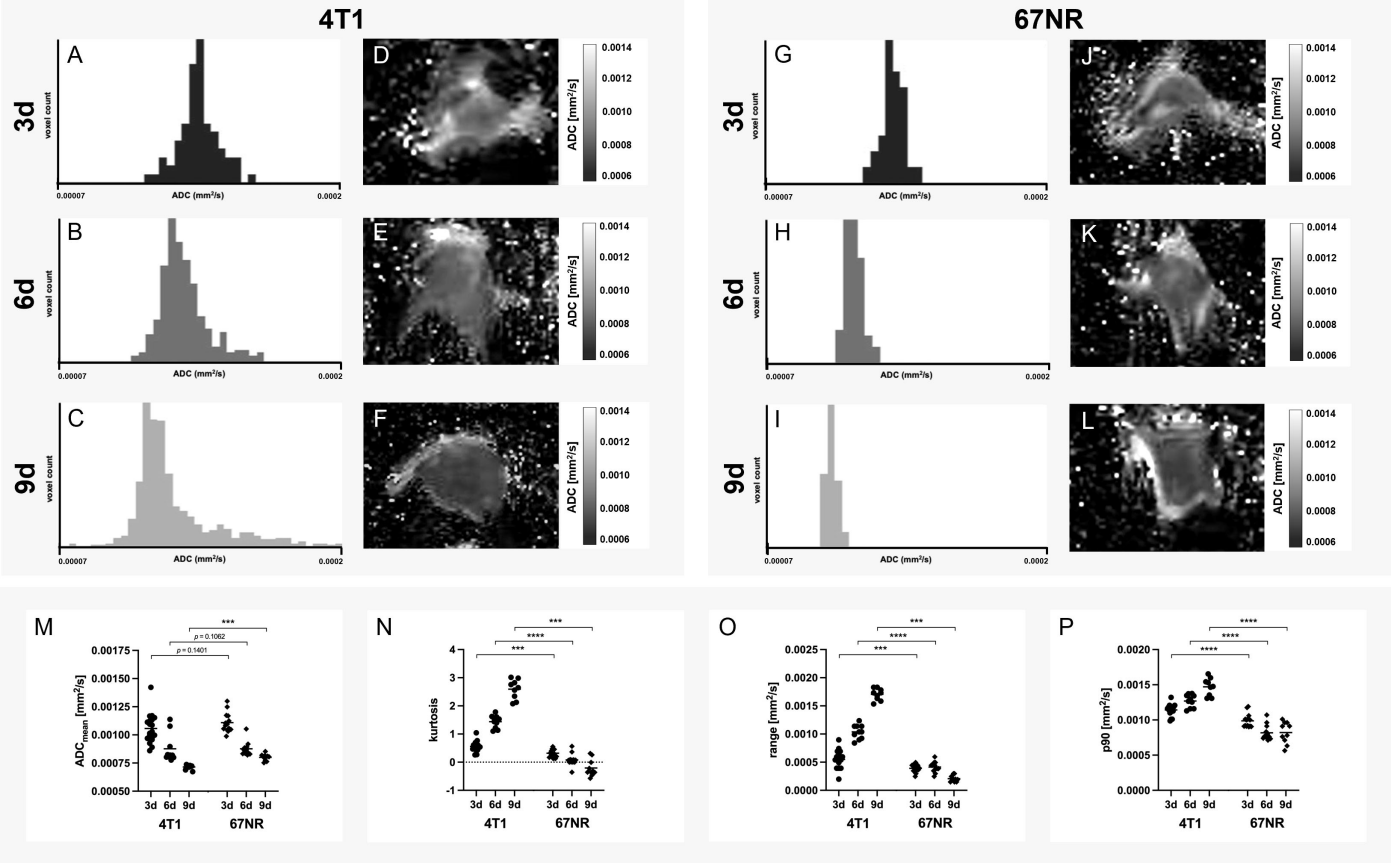
Histogram analysis of apparent diffusion coefficient. Exemplary histograms of 4T1 **(A–C)** and 67NR tumors **(G–I)**, as well as ADC maps (4T1 **D–F**, 67NR **J–L**) on days three, six and nine and the corresponding analyses **(M–P)**. Mean ADC was lower in 4T1 tumors than 67NR tumors **(M)**, while both tumor models showed decreasing values over time. ADC histogram kurtosis (sharpness of the peak of the frequency-distribution curve, N), range (difference between maximum and minimum value, **O**) and p90 (values above the 90^th^ percentile of all ADC values, **P**) all increased in 4T1 tumors over time and were higher than in 67NR tumors, which exhibited less pronounced decreasing values in the assessed parameters over time. This corresponds with tumoral structural heterogeneity, which can also be seen in the exemplary histograms, with a great range of values in 4T1 tumors **(A–C)** and a small range in 67NR tumors **(G–I)**. Mean values of the ROI covering the tumor in single slice imaging are shown. ***p < 0.001, ****p < 0.0001.

Mean ADC decreased for both tumor models over time, with higher values in the 67NR model in comparison to the 4T1 model at all time points, however not significant on days three and six (**Figure 2**, mean values, STD and p-values found in **Supplementary Table 1**). The values for histogram kurtosis, range and 90^th^ percentile (p90) were significantly higher in the 4T1 tumor model in comparison to the 67NR tumor model on day three and greatly increased until day nine. The 67NR model not only exhibited lower, but also slightly decreasing values during progression (**Figure 2**). Corresponding to the greater heterogeneity of the 4T1 tumors, these tumors exhibited a much wider range of ADC values, visible also in the exemplary histograms of 4T1 and 67NR tumors, and a higher histogram kurtosis, which increased as tumors progressed due to intratumoral structural changes such as increasing necrosis and hemorrhage. 67NR tumors were homogenous, as reflected in the overall lower values in the histogram analysis (kurtosis, range and p90). These tumors did not show larger necrotic areas at all of the three evaluated time points.

### 3.3 Native T1 and T2 mapping

**Figure 3:**
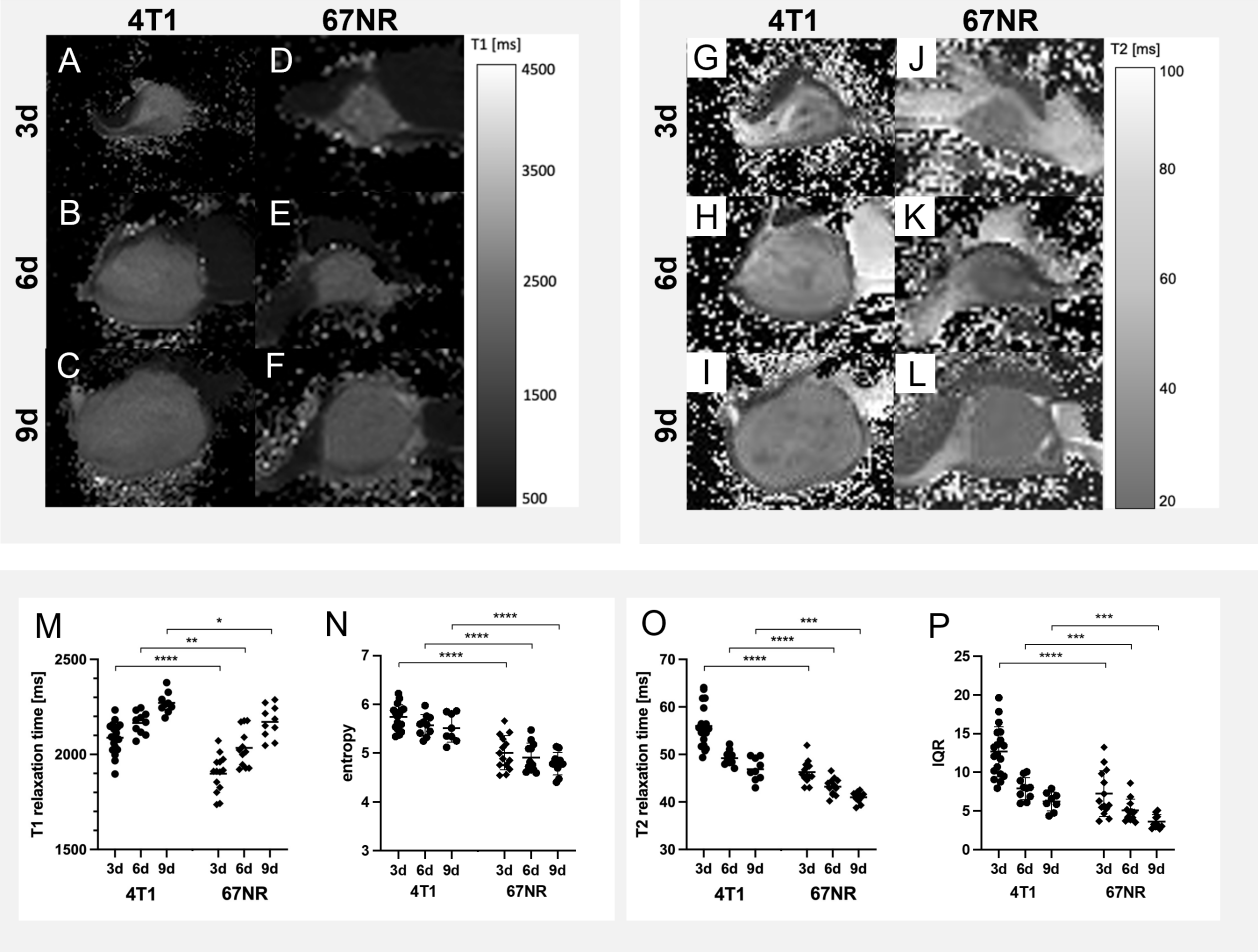
Exemplary imaging data and quantitative analyses of native T1 and T2 maps. The differences over time and between 4T1 **(A–C, G–I)** and 67NR **(D–F, J–L)** tumors are only slightly visible on T1 maps **(A–F)** and T2 maps **(G–L)**. The two tumor models however exhibited significantly different T1 and T2 values, but with similar changes during progression - while T1 relaxation times increased over time in both tumor models **(M)**, T2 times **(N)** decreased. Both T1 relaxation times **(M)** and T2 relaxation times **(O)** were higher in the 4T1 model than in the 67NR model. Further histogram analysis revealed higher values for the entropy of T1 maps in 4T1 tumors in comparison to 67NR tumors **(N)**, while entropy decreased during progression for both tumor models. The interquartile range of T2 maps was also higher in the high malignant 4T1 tumors in comparison to low malignant 67NR tumors **(P)**, with similarly decreasing values over time. Mean values of the ROI covering the tumor in single slice imaging are shown, details are found in **Supplementary Table 1**. *p < 0.05, **p < 0.01, ***p < 0.001, ****p < 0.0001.

T1 times were higher in 4T1 tumors than 67NR tumors at all time points, with an increase for both models during tumor growth. T2 relaxation times were also higher in the 4T1 tumors at all three time points, but with a decrease over time for both models, which was more pronounced in 4T1 tumors. In a further histogram analysis, entropy of T1 maps was higher in 4T1 tumors than in 67NR tumors, while it decreased slightly for both models during progression, which was not significant for either tumor model. The IQR of T2 maps was also higher in 4T1 tumors than 67NR tumors, with both tumors exhibiting a significant decrease of IQR values over time. Mean values are presented in the corresponding **Figure 3**, mean, STD and p-values can be found in **Supplementary Table 1**.

### 3.4 Dynamic contrast enhanced imaging

The accumulation of the contrast agent is an important indicator for vascular permeability, as the albumin-binding gadofosveset can only extravasate leaky vessels.

**Figure 4:**
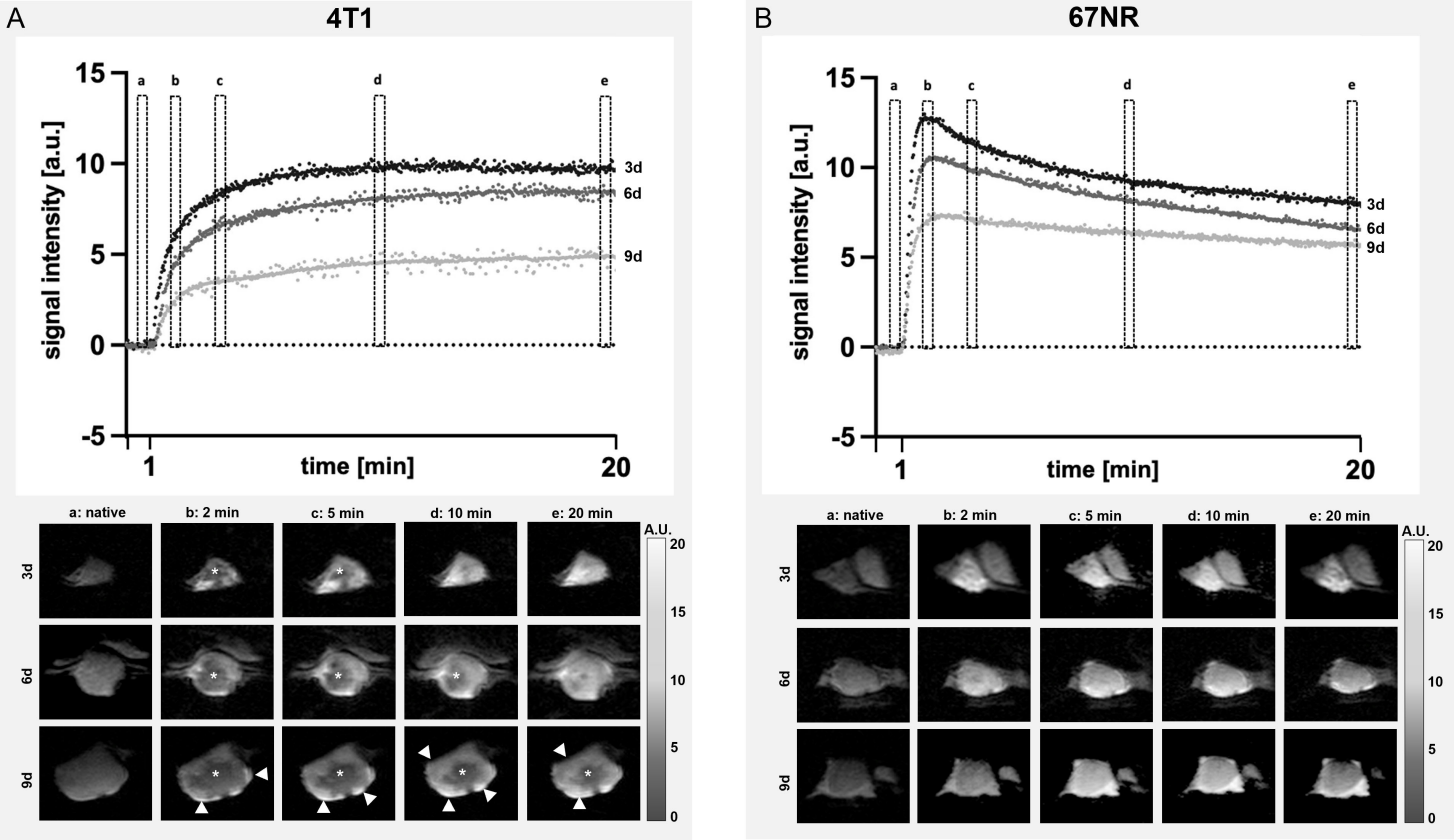
Assessment of contrast enhancement. Exemplary DCE curves of 4T1 **(A)** and 67NR **(B)** tumors on days three, six and nine and corresponding MR images show the differences in contrast enhancement, with a plateau in 4T1 **(A)** tumors, while 67NR **(B)** tumors exhibited a clear peak and a subsequent wash-out, which was more pronounced on day three in comparison to day nine. Images of the contrast enhancement of exemplary 4T1 and 67NR tumors show heterogeneous 4T1 tumors with a necrotic center (asterisks) and viable periphery (arrowheads), while 67NR tumors show a homogenous contrast agent enhancement. a–e are time points: a, native; b, 2 min; c, 5 min; d, 10 min; e, 20 min.

**Figure 5:**
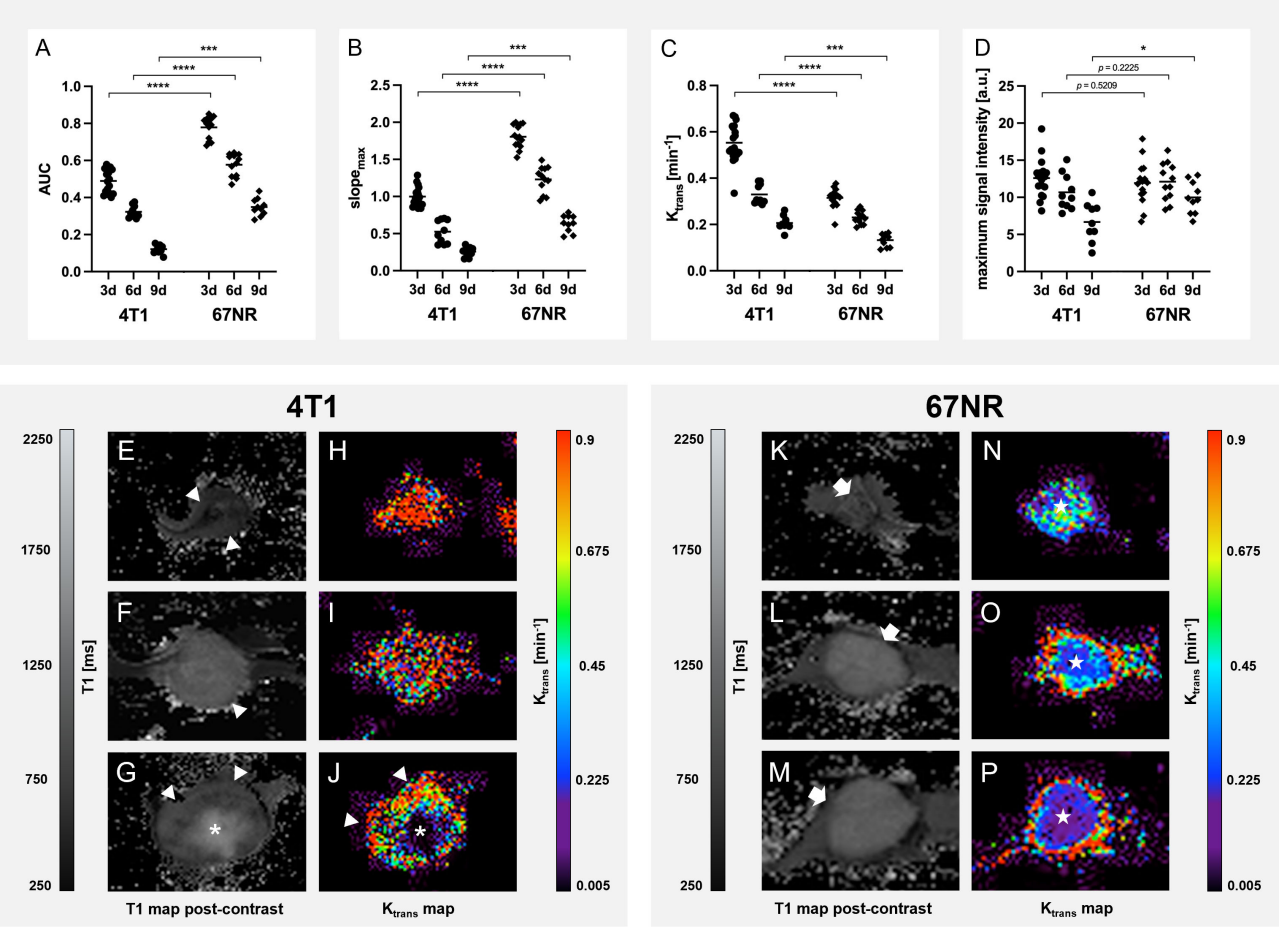
Detailed analysis of Dynamic Contrast-Enhanced imaging. Analysis of the DCE data with area under the curve (AUC, **A**), maximum slope(slopemax, **B**), Ktrans **(C)** and maximum signal intensity **(D)** show a higher permeability of 4T1 tumors than 67NR tumors, reflected in higher Ktrans values of 4T1 tumors, but with a greater inflow of the contrast agent in 67NR tumors, visible in AUC and slopemax, due to more intact blood vessels. Exemplary T1 and Ktrans maps of 4T1 tumors (**E–J**, respectively) and 67NR tumors (**K–P**, respectively) show heterogeneous areas of contrast agent retention with a viable periphery (arrowheads), and necrosis in the center (asterisks). All of the assessed parameters decreased over time, reflecting a reduced perfusion and permeability during progression for both tumor models. Mean values of the ROI covering the tumor in single slice imaging are shown. *p < 0.05, ***p < 0.001, ****p < 0.0001.

The contrast enhancement curves differed between 4T1 tumors and 67NR tumors (**Figure 4**), with a plateau for 4T1 tumors and markedly reduced maximum enhancement peak on day nine compared to day three for the 4T1 tumor. 4T1 tumors showed a heterogeneous enhancement, with viable areas in the periphery and a largely necrotic center. In the 67NR tumor model, contrast enhancement showed a high slope with an early peak and a slight wash-out of gadofosveset, which was less pronounced during tumor progression from day three to day nine. These tumors were largely homogeneous in DCE imaging, without central necrotic areas. The peak itself was not significantly different on days three and six between the two tumor models, but intratumoral changes in the 4T1 tumors on day nine led to a significantly reduced maximum intensity (**Figure 5**, details found in **Supplementary Table 1**).

K_trans_ values dropped over time in both tumor models and were higher in 4T1 tumors than in 67NR tumors, reflecting a greater permeability of 4T1 tumor vessels (**Figure 5**). Due to the more intact blood vessels in 67NR tumors, the enhanced perfusion led to higher values of AUC and slope_max_. During tumor progression, all of the assessed parameters decreased.

### 3.5 Analysis of contrast agent retention with MRI and laser ablation-inductively coupled plasma-mass spectrometry

**Figure 6:**
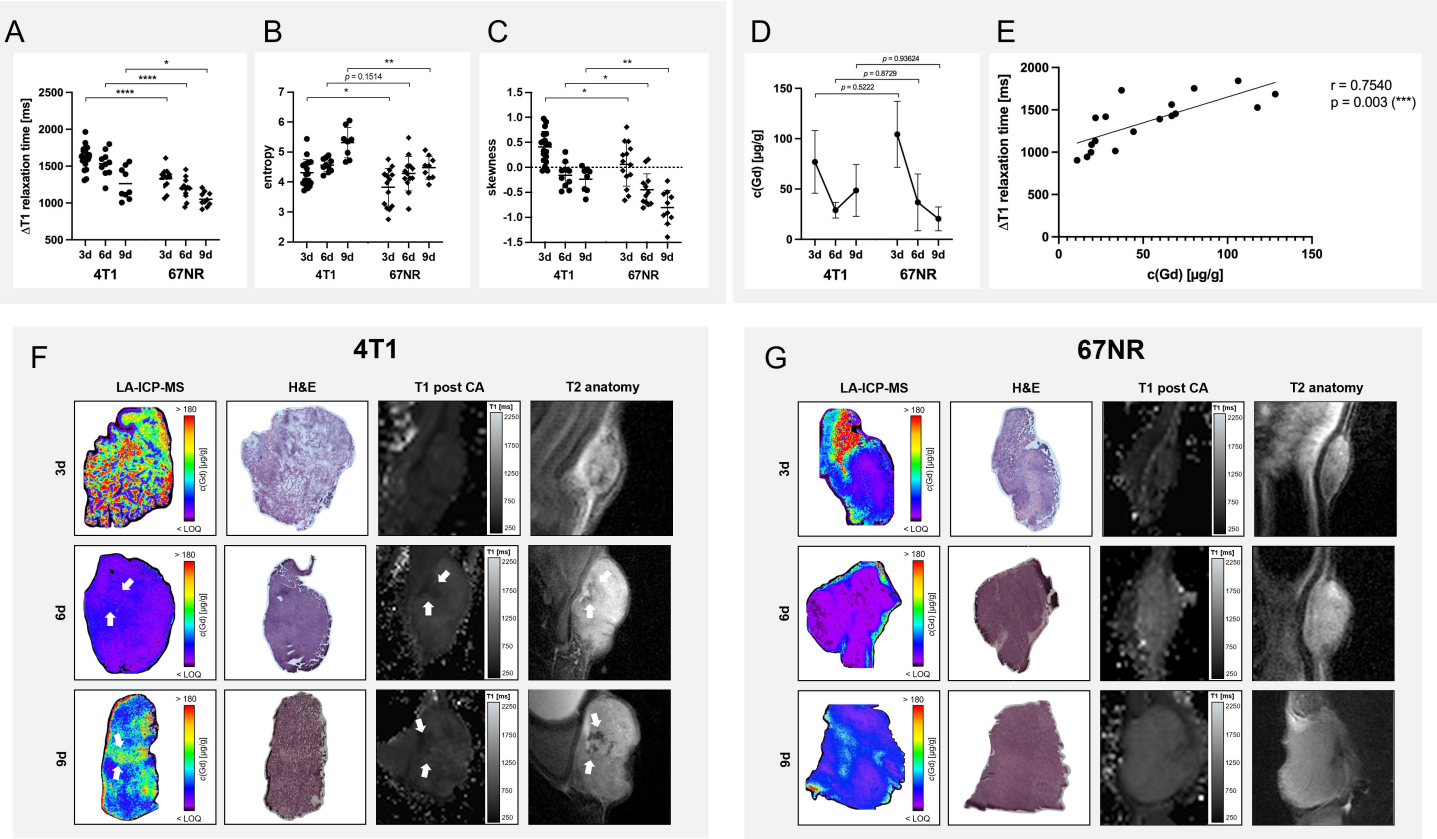
Analysis of contrast agent retention with MRI and LA-ICP-MS. Analysis of the delta T1 relaxation times **(A)** before and after injection of albuminbinding gadofosveset revealed a higher retention in 4T1 tumors in comparison to 67NR tumors, while both showed a decreasing accumulation of the contrast agent over time. Histogram analysis revealed a higher entropy (measure of gray-level distribution, **(B)** and skewness (asymmetry of probability distribution, **(C)** of 4T1 tumors than 67NR tumors. While the entropy increased during tumor progression for both tumor models, skewness decreased similarly for 4T1 and 67NR tumors. LA-ICP-MS showed a decreasing gadolinium retention in both tumor models over time **(D)**, with however non-significant differences between 4T1 and 67NR tumors, most likely due to intra- and intertumoral heterogeneity. Correlation between the values **(E)** showed a good correlation with significant results. Exemplary images of LA-ICP-MS, corresponding H&E and T2 and post-contrast T1 weighted MR images show a more heterogeneous distribution of gadolinium retention in 4T1 tumors (**F**, arrows), with necrotic areas that can be correlated between LA-ICP-MS, H&E and MRI, albeit not the exact same slices were analyzed. 67NR tumor were more homogeneous, without these focal necrotic areas **(G)**. Mean values of the ROI covering the tumor in single slice imaging are shown, details can be found in **Supplementary Table 1**. *p < 0.05, **p < 0.01, ***p < 0.001, ****p < 0.0001, CA – contrast agent.

Intratumoral contrast agent retention decreased during disease progression for both models, with more retention in the 4T1 tumors than in 67NR tumors, as assessed with T1 weighted MR imaging (**Figure 6**, detailed mean and p-values can be found in **Supplementary Table 1**). For assessing changes in T1 maps after injection of the contrast agent, the delta of T1 values before and after injection were calculated to eliminate the differences in pre-contrast T1 values. In the subsequent histogram analysis of T1 maps after injection of CM, 4T1 tumors showed higher values of entropy than 67NR tumors, albeit not significant on day six. In both models, the entropy increased significantly, which was more pronounced in the 4T1 tumor model (**Figure 6**; **Supplementary Table 1**). The values for skewness were also higher in 4T1 tumors than 67NR tumors, but decreased significantly during tumor progression for both models, from positive values on day three to negative values on day nine.

LA-ICP-MS results differed slightly from the MRI analysis, with non-significant differences between the two tumor models and a steeper decrease in contrast agent retention in 67NR tumors in comparison to 4T1 tumors (**Figure 6**). In more detail, this analysis revealed a gadolinium content of 77 ± 31 µg/g for 4T1 tumors vs. 104 ± 33 µg/g for 67NR tumors (p=0.7527) on day three, 29 ± 8 µg/g vs. 37 ± 28 µg/g (p=0.9986) on day six and 49 ± 26 µg/g vs. 20 ± 12 µg/g (p=0.0161) on day nine (**Figure 6**). A correlation analysis of the delta T1 relaxation times and the Gd concentration acquired by means of LA-ICP-MS showed a positive relationship between the variables (r=0.7540, p=0.003) (**Figure 6**). Areas of increased or decreased retention of Gd concentration in LA-ICP-MS also showed a different cellular composition in H&E imaging (necrotic areas) as well as in T2 weighted (hypointense areas) and contrast-enhanced T1 maps (**Figure 6**).

### 3.6 Histology and immunohistochemistry

**Figure 7:**
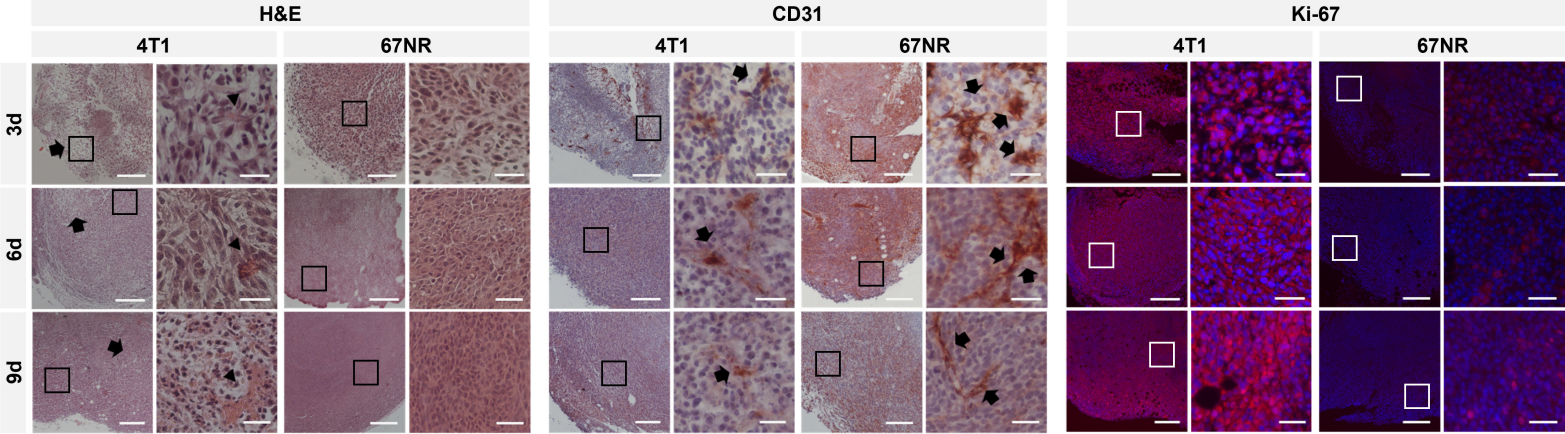
Histological and immunohistochemical analysis of tumor slices at 4x and 20x magnification. H&E staining showed heterogeneous 4T1 tumors with increasing intratumoral bleeding over time (arrowheads) and areas of necrosis (arrows), while 67NR were more homogenous and showed only little intratumoral hemorrhage. CD31 staining showed more CD31 positive cells (arrows) in 67NR tumors, which were also more organized. Fluorescence staining for Ki67 revealed a higher expression in 4T1 tumors than 67NR tumors on days three, six and nine (stained in red, DAPI in blue), reflecting the highly malignant phenotype. In 4T1 tumors, the expression seemed to increase, while in 67NR tumors, the expression slightly decreased. Scale bars represent 200 μm (4x magnification) and 50 μm (20x magnification). Boxes in the 4x magnification images represent the area of the 20x magnification images.

Different histological stainings were conducted to validate the observed changes in MRI (**Figure 7**). H&E staining verified increasing intratumoral hemorrhage and necrosis in 4T1 tumor over time, while 67NR tumors had only little intratumoral bleeds and small areas of necrosis, if any. As expected, Ki67 showed a much higher expression in 4T1 tumors, which seemed to increase over time, while 67NR tumors showed a lower expression, which, visually assessed, slightly decreased during progression. To analyze the vascular composition of the tumor, which was assessed with the injection of the contrast agent in the MRI examination, endothelium staining using CD31 staining was performed. CD31 showed a higher expression in 67NR tumor compared to deformed vessels in 4T1 tumors. In both tumor models, expression of CD31 decreased during tumor progression.

### 3.7 Electron microscopy

**Figure 8:**
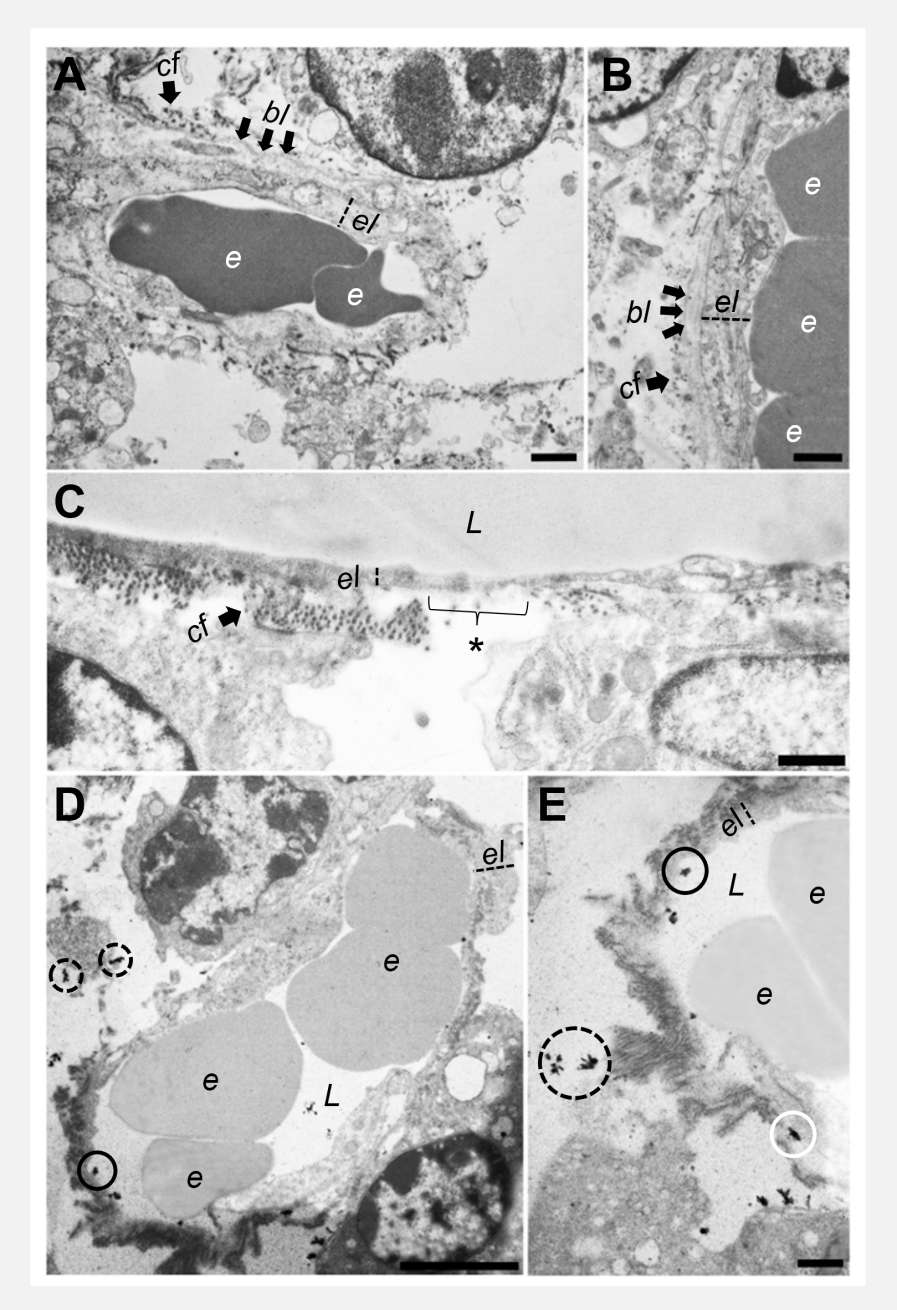
Transmission electron microscopy of endothelial cells. 67NR tumor vessels **(A, B)** show intact and continuous endothelial layers with an uninterrupted basal lamina and collagen fibrils on the outside. 4T1 tumors **(C)** show overall thinner endothelial layers with interruptions of the basal lamina and the collagenous layer (asterisk). Representative images of 4T1 tumors after gadofosveset injection **(D, E)** show the presence of electron dense contrast agent in the lumen of the vessels (black circles), between endothelial cells and basal lamina (white circle), and additionally, extravasated outside of the vessels (dashed circles). Scale bars represent 1 μm. *e = erythrocyte, el = endothelial layer, bl = basal lamina, cf = collagen fibrils, L = lumen*.

Transmission electron microscopy was used to investigate the ultrastructural integrity of the microvascular endothelial cells in both tumor models. The microvascular system of 67NR tumors was characterized by a continuous layer of endothelial cells connected by cell-cell contacts and surrounded with an intact basal lamina followed by a collagenous layer. In contrast, the integrity of the endothelium of 4T1 tumor vessels was severely altered: the endothelial cells were partly detached from the basal lamina and endothelial morphology including the cellular shape was changed. Moreover, cell-cell contacts were incomplete or absent and the continuous layer of endothelial cells was partly disrupted. The injected contrast agent was able to extravasate and was found outside of the vessels, supporting the compromised integrity in highly malignant 4T1 tumors (**Figure 8**).

### 3.8 Principal component analysis

**Figure 9:**
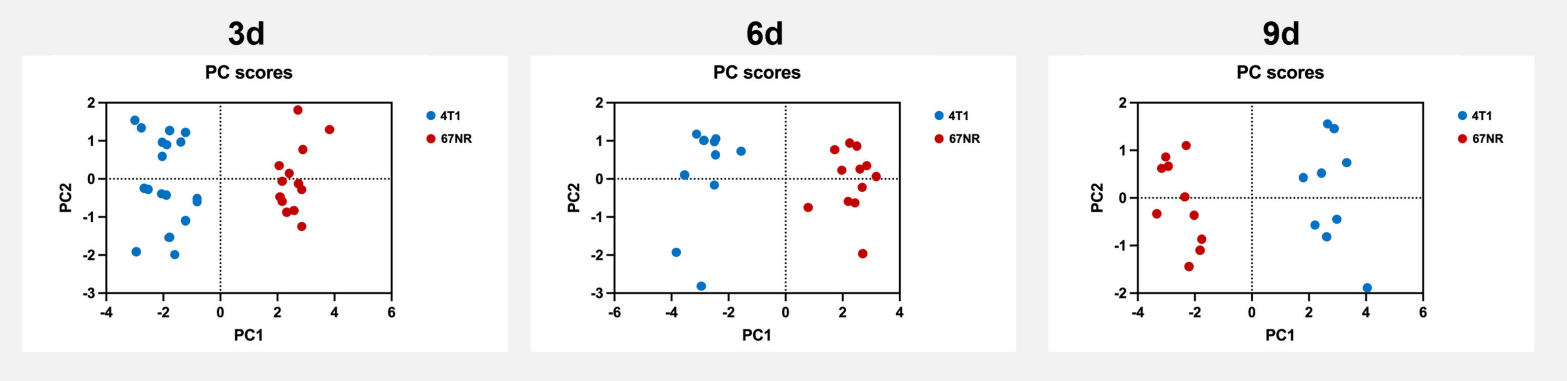
Principal component analysis score plots of the assessed multiparametric MRI data. Principal component analysis score plots revealed a clear discrimination of the highly malignant metastatic 4T1 tumors and the non-metastatic 67NR tumors, based on the presented multiparametric MRI data. Shown are the first two principal components (PC1 and PC2). The first principal component (PC1) already explained 56.5% (3d), 71.4% (6d) and 76.5% (9d) of the total variance. Detailed values can be found in **Supplementary Tables 2–4**.

The ten calculated principal components based on the variables ADC_mean_, ADC histogram kurtosis, ADC histogram range, ADC histogram p90, ΔT1 relaxation time, K_trans_, AUC, slope_max_, T1 relaxation time and T2 relaxation time explained 100% of the total variance (**Figure 9**). Thus, the two tumor models can be distinguished with the presented multiparametric MRI protocol. The weights of the original variables on each component (loadings) are shown in **Supplementary Tables 2–4**.

## 4 Discussion

Tumor heterogeneity is a major challenge of modern oncological therapy, as it can adversely affect clinical outcome and is one of the main causes of failure in cancer therapy (2). However, evaluation of heterogeneity by regular histology after biopsy is not appropriate and a high sampling bias can affect the results. Multiparametric MRI by simultaneous assessment of different tumoral features, which can be repeated regularly under therapy, can overcome this challenge.

The used multiparametric MRI revealed significant changes in tumor heterogeneity and structural composition during tumor progression, as well as differences between highly metastatic 4T1 tumors and the non-metastatic 67NR tumor model. Detailed knowledge of individual tumor biology constitutes the prerequisite for personalized treatment decisions, optimized for certain tumor characteristics such as altered endothelial permeability (4). Some of the applied techniques are established in clinical routine, such as diffusion-weighted imaging. To facilitate MRI-based tumor assessment and highlight the advantages of a multi-parameter scan routine, we have constructed a one-stop-shop assessment for a variety of tumor features addressing tumor heterogeneity in two tumor models with different degrees of malignancy over time during tumor progression.

When tumors increase in size during progression, the lack of sufficient blood supply leads to intratumoral hypoxia with subsequent necrosis. Already after three days, central necrotic areas became visible in 4T1 tumors in T2-weighted images, representing a more malignant phenotype with features suggestive of intratumoral hypoxia resulting from a faster tumor growth (24). This intratumoral hypoxia stimulates neoangiogenesis and the formation of distorted, unorganized blood vessels, while slower growing 67NR tumors form blood vessels with features closer to physiological blood vessels (25, 26). These differences can also be observed during tumor progression, as blood supply to 67NR tumors seems sufficient, with only negligible areas of intratumoral necrosis, while the unorganized blood vessels of 4T1 tumors led to an increasing development of necrotic areas over time. This distinct intratumoral vasculature is one of the most important features to evaluate, as vascular leakage is a key characteristic of highly malignant tumors and can be addressed specifically with targeted therapy. Gadofosveset enables to specifically assess tumor vessel leakage as albumin-binding gadolinium molecules extravasate through the distorted endothelium into the vessel wall and the interstitium (27, 28). Since it can thus extravasate from immature blood vessels of highly malignant tumors, it detects angiogenesis efficiently and shows different properties than the extracellular gadobutrol (29). After injection of the contrast agent, the fast increase in signal intensity of 67NR tumors, represented by the peak enhancement, but also by a high maximum slope and area under the curve, in comparison to 4T1 tumors, can be attributed to the more intact blood vessels in 67NR tumors that enable the fast bolus transport of the contrast agent into and out of the tumor (30, 31). This was reflected by a thicker endothelial layer of vessels in 67NR tumors, while 4T1 tumors showed a disrupted and thin endothelial layer in electron microscopy. Furthermore, a higher number of CD31 positive endothelial cells in 67NR tumors that form vascular structures were visible, whereas these were fewer and more unorganized in 4T1 tumors. This observation has also been found in a study evaluating tumor perfusion with 2-^18^F-Fluorethanol in these two tumor models, distinguishing areas of good and poor perfusion and correlating those with the CD31 expression (32). In both tumor models, the expression of CD31 decreased during progression, which has also been found in a study by Serganova et al. (26). The leaky blood vessels of 4T1 tumors result in a higher degree of permeability reflected in higher K_trans_ values, which was similarly found in a study evaluating the role of retinol-binding protein 4 in the metastatic potential of breast cancer, also using 4T1 and 67NR as tumor models (30). Thus, while inflow of the contrast agent was reduced in 4T1 tumors, extravasation was enhanced with albumin-binding gadofosveset, which extravasates through leaky vessel walls and is not transferred back into the intravascular space (33). The more heterogenous phenotype of 4T1 tumors is reflected in K_trans_ maps as well, with higher values in the periphery and lower values in the necrotic tumor center, in comparison to largely homogenous K_trans_ maps of 67NR tumors (32). This was also found in a study evaluating 4T1 and 67NR tumors with second harmonic generation imaging and resonance Raman spectroscopy (34). Corresponding to the decreasing CD31 expression and increasing areas of intratumoral necrosis, markers of tumor perfusion and permeability decreased during progression for both tumor models. However, K_trans_ does not enable to fully quantify the extravasated Gadolinium in the interstitial space, which was achieved with LA-ICP-MS in this study. A lower retention in 67NR at all time points was expected in the LA-ICP-MS results, however, while the two tumor models have characteristic features, there is still a significant inter-and intratumoral heterogeneity. This could be the reason for the difference in contrast agent accumulation in LA-ICP-MS, with the lower gadolinium retention on day six in comparison to day nine in 4T1 tumors (34). In the beforementioned study by Bendau et al., an accurate discrimination between 4T1 and 67NR tumors was possible, but the authors also had some misdiagnoses inter alia due to intertumoral heterogeneity. However, an overall decreasing retention of the contrast agent over time was found with MRI and LA-ICP-MS alike, resulting in a positive correlation between the two methods. DCE imaging has been widely used for diagnosis and staging of cancer, as well as tumor response, especially to therapies targeting vascular endothelial growth factor, but is rarely performed in clinical routine to date (35). It has furthermore already proven to correlate with histology regarding different sub-regions in the tumor differentiating normoxic from hypoxic regions in 4T1 and 67NR tumors (10). The combination of DCE and LA-ICP-MS in this study enabled to quantify the retained contrast agent with a high temporal (DCE) and spatial (LA-ICP-MS) resolution as a marker for endothelial dysfunction. The further histogram analysis of T1 maps after injection of gadofosveset for markers of heterogeneity also revealed a higher entropy, which is a measure for the gray-level distribution (36) or “irregularities” of the histogram (37) and skewness, which is a measure of asymmetry of probability distribution, in 4T1 than 67NR tumors. A higher skewness and a trend towards a higher entropy has been associated with a higher risk of disease recurrence and thus a worse outcome in patients with breast cancer (38). An increasing entropy for both models was found, reflecting an increasingly heterogeneous distribution of the retained contrast agent. The decreasing skewness seems to be reflective of tumor progression in our study.

The distinction of highly and low-malignant tumors however cannot be based on vascular features alone, making a detailed analysis of the structural composition necessary. The two tumor models also differ in cellularity, with a homogenous cell composition in 67NR tumors, mainly consisting of dense tumor cells also during progression, in comparison to the more heterogenous cellular composition of 4T1 tumors, consisting of less dense tumor cells, with increasing necrotic areas in between. Diffusion weighted imaging with different b-values and subsequent calculation of the apparent diffusion coefficient (ADC) enables to visualize differences in cellularity and structural composition (39, 40). While mean ADC is a summation of several effects, histogram analyses focus on specific calculated parameters that have been found to enable differentiation of high-grade and low-grade tumors due to differences in structural composition of the tumors, inter alia determination of the intratumoral spatial heterogeneity in xenograft breast cancer models (41, 37). Range reflects the distribution of ADC values within the tumor and histogram kurtosis quantifies the deviation from the Gaussian form. A high degree in cellularity leads to a hindered diffusion of water molecules and lower ADC values which is typically found in highly malignant tumors (42, 43). Further analysis of histogram kurtosis enabled for differentiation of different grades in patients with gastric tumors (44), as well as between low and high grade tumors in patients with renal cell carcinoma (45), although it has not been used in preclinical studies. Higher histogram kurtosis and range values, as found in 4T1 tumors, indicate more heterogeneous internal components and poorer cell differentiation, which was found to inversely correlate with the prognosis in patients with hepatocellular carcinoma (46) and aided in differentiation of ER-positive and triple negative subtypes in breast cancer patients (47). Although these changes were found in clinical studies and thus cannot be transferred to our preclinical data, the found changes were similar to our high and low malignant tumor models. Furthermore, the heterogeneity increased over time in 4T1 tumors, as reflected inter alia in a wider range and kurtosis of the histogram analysis and contrary, the heterogeneity of 67NR tumors as assessed with analysis of diffusion-weighted imaging did not increase during tumor progression. These features of tumoral composition correlate with native T1 times as well, which increase with the amount of necrotic areas and were thus higher in high malignant than low malignant tumors (48), as confirmed here. In the clinical setting examining patient with kidney tumors, native T1 times enabled to differentiate high grade clear cell renal cell carcinoma (cRCCs) from lower grade cRCCs (49). The results also show the increase in T1 values during tumor progression (50), likely due to areas of dense proliferative undifferentiated tumor cells (51, 52) and increasing density of the extracellular matrix and collagen contents, assessed in a rabbit hepatic cancer model (48) and in specimens of breast cancer patients (53). However, in some studies, including a study evaluating the effect of anti-angiogenic therapy in a mouse ovarian cancer model, T1 times did not change during tumor growth (51). A further analysis of T1 maps revealed higher values of 4T1 tumors than 67NR tumors for entropy, which reflects the irregularity of gray-level distribution (36) and has been associated with the amount of chaos in a system (54). The slight decrease over time for both models might be due to the differing sizes of the ROIs, which has been found as an influence of entropy in patients with metastases of different solid tumors (55). Although these findings were analyzed in the clinical setting, the ROIs in our study differed in their size during tumor progression as well, as they covered the entire tumor volume. The IQR of T2 maps has been used as a measurement of heterogeneity in mouse models of pancreatic cancer, with a higher IQR reflecting a more heterogeneous tumoral composition (56). The higher IQR of 4T1 tumors due to larger areas of necrosis in comparison to 67NR tumors is in line with these results, the decreasing values for both tumor models might be due to the volume share of necrotic areas in comparison to the tumor volume.

Mapping techniques enable to quantify T1 and T2 relaxation times and assess intratumoral hemorrhage, necrosis and edema (57). These phenomena influence the signal intensities in different ways, iron and hemorrhage were found to mainly decrease T1 and T2 times, while inflammation and necrosis were found to increase these parameters (57). In more detail, intratumoral edema with an increased fluid content can lead to an increase in tumor size and can typically be observed after tumors have reached a diameter of 3 mm (58). During disease progression, larger areas of edema were visible, but their volume share decreased in relation to total tumor volume, which likely contributed to the observed decreased T2 relaxation times (59, 60). The dense tumor cells of 67NR tumors also lead to lower T2 times, corresponding to results for the experimental pancreas tumors BXPC3 und Panc02, which similarly have differing grades of malignancy (56).

The effects of intratumoral hemorrhage on MRI signals can differ, depending on the age of the hemorrhage, but most likely contributed to the decrease in T2 and the increase in T1 relaxation times (57). 4T1 tumors exhibited increasing intratumoral hemorrhage due to vascular leakiness over time, while 67NR tumors were largely homogenous. However, the observed changes in MRI might be similar to the changes in certain pathological features, as assessment of the changes in diffusion-weighted imaging has already been found to correlate with the Ki67 proliferation status in a mouse model of rhabdomyosarcoma (61) and has been found useful to predict histological markers such as Ki67 in the breast cancer xenografts MCF-7 and MDA-MB-231 (62). Although only visually assessed in this study, 4T1 tumors exhibited a higher Ki67 proliferation status overall, with an increase over time and 67NR tumors had a lower Ki67 index overall and a decrease over time, which was also found in the p90 histogram analysis.

Overall evaluation of the results with principal component analysis revealed a consistently high differentiability of the malignancy grades of the two tumor models with the presented imaging protocol.

As a limitation, the MRI sequences were performed in single slice mode, which did not allow for full tumor coverage and led to loss of information in comparison to 3D tumor coverage. Still, initial multislice T2w RARE images suggested that representative slices from the center of mass of the individual tumor were chosen and this assessment was valid for all tumor stages. Additionally, the applied contrast agent gadofosveset is not in clinical routine any longer, so access is limited. In the presented study, only two tumor models of differing malignancy grades were compared, limiting the translation to other tumor models and clinical studies. Furthermore, we have included different MRI parameters to assess certain features of malignancy, however, there are a variety of other MRI sequences that assess tumoral features, e.g. creatine CEST MRI, that we have not included in our protocol (63).

In conclusion, the presented multiparametric imaging protocol combines established imaging techniques with state-of-the-art analyses to assess a variety of different tumor features, which might enable non-invasive differentiation of different degrees of malignancy.

## Data availability statement

The original contributions presented in the study are included in the article/**Supplementary Material**. Further inquiries can be directed to the corresponding author.

## Ethics statement

The animal study was reviewed and approved by state agency for nature, environment and consumer protection of North Rhine-Westfalia, Germany, (LANUV; approval ID 81-02.04.2018.A010).

## Author contributions

The author contributions were as followed: MG – conceptualization, methodology, investigation, writing – original draft, editing; EH – investigation, methodology, original draft preparation, writing- review and editing; KK – investigation (LA-ICP-MS); UH – investigation (electron microscopy); MM – validation, data curation; AH – writing – review and editing; CG – investigation, animal care; LW – investigation; CH – investigation; BM – investigation; VH – software; TK – investigation (software); LH – investigation (LA-ICP-MS); WH – supervision; UK – supervision (LA-ICP-MS), MK – investigation (histology/immunohistochemistry); RS – investigation (statistics); ME – supervision, writing – review and editing; CF – supervision, writing – review and editing; MW – supervision, writing – review and editing. All authors contributed to the article and approved the submitted version.

## Funding

This study was supported by the German Research Foundation (DFG, grant no. GE 3336/1-1), the Dean’s office of the faculty of medicine, University of Münster (fellowship to M.G.), the medical faculty of the University of Münster (MedK, fellowship to E.H.) and the IZKF Münster - PIX. The LA-ICP-MS part of this work was funded by the German Research Foundation (DFG, grant CRC1450 – 431460824, to U.K.).

## Acknowledgments

Technical support from Klaudia Niepagenkemper, Claudia Terwesten-Solé and Richard Holtmeier is gratefully acknowledged. We acknowledge support from the Open Access Publication Fund of the University of Muenster.

## Conflict of interest

The authors declare that the research was conducted in the absence of any commercial or financial relationships that could be construed as a potential conflict of interest.

## Publisher’s note

All claims expressed in this article are solely those of the authors and do not necessarily represent those of their affiliated organizations, or those of the publisher, the editors and the reviewers. Any product that may be evaluated in this article, or claim that may be made by its manufacturer, is not guaranteed or endorsed by the publisher.

## Abbreviations

ADC, apparent diffusion coefficient; DCE, dynamic contrast-enhanced; DFG, Deutsche Forschungsgemeinschaft, German Research Foundation; DMEM, Dulbecco’s modified eagle medium; DWI, diffusion weighted imaging; DW-EPI, diffusion weighted echo planar imaging; FLASH, fast low angle shot; FOV, field of view; LA-ICP-MS, laser ablation-inductively coupled plasma-mass spectrometry; LANUV, Landesamt für Natur, Umwelt und Verbraucherschutz (state agency for nature, environment and consumer protection); MRI, magnetic resonance imaging; PCA, principal component analysis; RARE, rapid acquisition with relaxation enhancement; ROI, region of interest; STD, standard deviation; TE, echo time; TR, repetition time.
