# Supplementary material for: Multiparametric MRI enables for differentiation of different degrees of malignancy in two murine models of breast cancer
Source: Front Oncol. 2022 Nov 2;12:1000036. doi: 10.3389/fonc.2022.1000036 (PMC9667047; doi:10.3389/fonc.2022.1000036)
Supplement: Supplementary file 1 [file Table_1.docx]

|  | **Descriptive statistics** | | | | | | **Analysis of normal distribution** | **Comparison between both**  **tumor models** | | | **Analysis of tumor progression** | |
| --- | --- | --- | --- | --- | --- | --- | --- | --- | --- | --- | --- | --- |
| **Parameter** | **Mean + STD** | | | | | | ***p*-value** | ***p*-value** | | | ***p*-value** | ***p*-value** |
|  | 4T1 3d | 4T1 6d | 4T1 9d | 67NR 3d | 67NR 6d | 67NR 9d | Shapiro-Wilk | 3d: 4T1 vs. 67NR | 6d: 4T1  vs. 67NR | 9d: 4T1 vs. 67NR | 4T1 3d 🡪 6d  🡪 9d | 67NR 3d 🡪 6d 🡪 9d |
| volume [mm^3^] | 13.75 ± 3.72 | 46.94 ± 12.73 | 113.65 ± 33.96 | 5.31 ± 2.09 | 18.41 ± 4.75 | 50.18 ± 13.23 | <0.0001 | <0.0001 | <0.0001 | 0.0003 | <0.0001 | <0.0001 |
| T1 time [ms] | 2119.7 ± 188.2 | 2165.7 ± 58.2 | 227.01 ± 55.2 | 1899.0 ± 98.0 | 2034.7 ± 99.0 | 2171.1 ± 84.7 | 0.0009 | <0.0001 | 0.0041 | 0.0160 | <0.0001 | <0.0001 |
| T1 entropy | 5.74 ± 0.25 | 5.57 ± 0.22 | 5.51 ± 0.29 | 5.01 ± 0.35 | 4.91 ± 0.3 | 4.78 ± 0.23 | 0.0334 | <0.0001 | <0.0001 | <0.0001 | 0.0809 | 0.3480 |
| T1 entropy GD | 4.3 ± 0.45 | 4.57 ± 0.22 | 5.32 ± 0.51 | 3.83 ± 0.63 | 4.28 ± 0.58 | 4.48 ± 0.38 | 0.1720 | 0.0158 | 0.1514 | 0.0015 | <0.0001 | 0.0228 |
| T1 skewness GD | 0.41 ± 0.31 | -0.16 ± 0.25 | -0.24 ± 0.24 | 0.06 ± 0.44 | -0.45 ± 0.33 | -0.8 ± 0.34 | 0.8961 | 0.0114 | 0.03 | 0.0011 | <0.0001 | <0.0001 |
| T2 time [ms] | 55.98 ± 4.41 | 49.23 ± 1.53 | 46.88 ± 2.41 | 46.27 ± 2.33 | 43.22 ± 1.80 | 40.97 ± 1.23 | <0.0001 | <0.0001 | <0.0001 | 0.0003 | <0.0001 | <0.0001 |
| T2 IQR | 12.7 ± 3.22 | 7.91 ± 1.42 | 6.23 ± 1.22 | 7.25 ± 2.95 | 5.07 ± 1.47 | 3.64 ± 0.9 | 0.0002 | <0.0001 | 0.0003 | 0.0005 | <0.0001 | 0.0005 |
| ADC_mean_ [mm^2^/s] | 0.0011 ± 0.0001 | 0.0009 ± 0.0001 | 0.0007 ± 0.00001 | 0.0011 ± 0.0001 | 0.0009 ± 0.0001 | 0.0008 ± 0.00001 | <0.0001 | 0.1401 | 0.1062 | 0.0003 | <0.0001 | <0.0001 |
| kurtosis | 0.56 ± 0.18 | 1.44 ± 0.22 | 2.59 ± 0.35 | 0.32 ± 0.14 | 0.08 ± 0.22 | -0.21 ± 0.29 | <0.0001 | 0.0009 | <0.0001 | 0.0003 | <0.0001 | <0.0001 |
| range [mm^2^/s] | 0.0005 ± 0.0002 | 0.001 ± 0.0001 | 0.0017 ± 0.0001 | 0.0004 ± 0.0001 | 0.0004 ± 0.0001 | 0.0002 ± 0.0001 | <0.0001 | 0.00045 | <0.0001 | 0.0003 | <0.0001 | <0.0001 |
| p90 [mm^2^/s] | 0.0011 ± 0.0001 | 0.0013 ± 0.0001 | 0.0015 ± 0.0001 | 0.0010 ± 0.0001 | 0.0008 ± 0.0001 | 0.0008 ± 0.0001 | 0.4393 | <0.0001 | <0.0001 | <0.0001 | <0.0001 | 0.001 |
| AUC | 0.49 ± 0.06 | 0.32 ± 0.03 | 0.12 ± 0.02 | 0.78 ± 0.06 | 0.58 ± 0.06 | 0.35 ± 0.05 | 0.0200 | <0.0001 | <0.0001 | 0.0003 | <0.0001 | <0.0001 |
| slope_max_ | 0.99 ± 0.13 | 0.52 ± 0.15 | 0.26 ± 0.07 | 1.80 ± 0.15 | 1.23 ± 0.18 | 0.64 ± 0.11 | 0.0345 | <0.0001 | <0.0001 | 0.0003 | <0.0001 | <0.0001 |
| K_trans_ [min^-1^] | 0.55 ± 0.08 | 0.33 ± 0.04 | 0.21 ± 0.03 | 0.31 ± 0.04 | 0.23 ± 0.03 | 0.13 ± 0.03 | <0.0001 | <0.0001 | <0.0001 | 0.0007 | <0.0001 | <0.0001 |
| max. intensity [a.u.] | 12.59 ± 2.62 | 10.67 ± 2.39 | 6.67 ± 2.64 | 11.92 ± 2.91 | 12.11 ± 2.55 | 9.98 ± 2.15 | 0.9791 | 0.5209 | 0.2225 | 0.0106 | <0.0001 | 0.1255 |
| ΔT1 [ms] | 1618.0 ± 162.6 | 1532.8 ± 172.0 | 1262.0 ± 215.0 | 1329.3 ± 142.8 | 1194.0 ± 142.9 | 1051.6 ± 96.5 | 0.3096 | <0.0001 | <0.0001 | 0.0208 | <0.0001 | <0.0001 |
| c(Gd) [µg/g] | 76.92 ± 31.15 | 28.94 ± 7.75 | 48.60 ± 25.77 | 104.30 ± 32.77 | 36.77 ± 28.15 | 20.40 ± 11.78 | 0.0132 | 0.5222 | 0.8729 | 0.9362 | <0.0001 | <0.0001 |

**Supplementary table 1.** Descriptive and analytical statistics of all scan parameters.

Shapiro-Wilk test was used to analyze data distribution; for *p*-value ≤ 0.05, Mann-Whitney U test (comparison between both tumor models) and Kruskal-Wallis test (analysis of tumor progression) were conducted, for *p*-value > 0.05, unpaired t-test and one-way ANOVA were performed.

|  | **3d** | | | | | | | | | |
| --- | --- | --- | --- | --- | --- | --- | --- | --- | --- | --- |
| **Variable** | **PC1** | **PC2** | **PC3** | **PC4** | **PC5** | **PC6** | **PC7** | **PC8** | **PC9** | **PC10** |
| ADC_mean_ | -0.768231398 | 0.189321768 | -0.229339698 | 0.40378766 | -0.006407946 | 0.164327012 | 0.361344488 | 0.004994191 | 0.024387595 | 0.010131659 |
| kurtosis | -0.84139206 | 0.064451353 | 0.256310799 | 0.252567159 | 0.173666142 | 0.021157161 | -0.149766904 | 0.309060339 | -0.032376135 | -0.093892715 |
| range | 0.415910203 | 0.796584998 | -0.059111428 | 0.001739262 | 0.421934843 | -0.050263633 | -0.048045466 | -0.071614528 | -0.016494341 | 0.026633476 |
| p90 | -0.850440365 | 0.036126013 | 0.383537256 | -0.033111099 | -0.017710673 | 0.115033525 | -0.010795485 | -0.225537832 | -0.245114554 | -0.051357029 |
| ΔT1 time | 0.934632872 | 0.023389381 | -0.123261004 | -0.158875432 | 0.020276637 | 0.018024895 | 0.152171318 | 0.021361008 | -0.037115891 | -0.244445542 |
| K_trans_ | 0.926421398 | 0.00805885 | 0.02571262 | 0.002764507 | -0.06450995 | -0.05437629 | 0.13047619 | 0.193991904 | -0.260226783 | 0.107314856 |
| AUC | -0.744499643 | -0.267039145 | -0.361458296 | 0.048333558 | 0.192032137 | -0.439632388 | 0.043216587 | -0.047334655 | -0.081104025 | -0.024146636 |
| slope_max_ | -0.590108427 | 0.587232316 | -0.006525352 | -0.164087079 | -0.484048705 | -0.204499076 | 0.000522963 | 0.05536343 | 0.01011728 | -0.025929505 |
| T1 time | -0.595155846 | 0.024371388 | -0.67808888 | -0.301298717 | 0.00754432 | 0.253382277 | -0.134987509 | 0.061333868 | -0.091182493 | 0.007272962 |
| T2 time | -0.683138549 | -0.046821489 | 0.285314317 | -0.58493629 | 0.214775583 | 0.001281135 | 0.217481997 | 0.093529493 | 0.062960698 | 0.037872443 |

**Supplementary table 2.** Weights of the original variables on each calculated principal component (loadings) on day 3.

|  | **6d** | | | | | | | | | |
| --- | --- | --- | --- | --- | --- | --- | --- | --- | --- | --- |
| **Variable** | **PC1** | **PC2** | **PC3** | **PC4** | **PC5** | **PC6** | **PC7** | **PC8** | **PC9** | **PC10** |
| ADC_mean_ | -0.605134953 | 0.46688725 | -0.585986889 | 0.158393811 | -0.211519351 | -0.000535546 | -0.048469909 | 0.014237951 | -0.008122568 | -0.000057 |
| kurtosis | -0.911498921 | -0.137687537 | 0.036547376 | 0.242404197 | 0.040113815 | 0.155839594 | 0.249927118 | -0.039884734 | -0.006759159 | 0.011009706 |
| range | -0.053262124 | -0.956783176 | -0.221378316 | 0.113207172 | -0.106772001 | -0.008606614 | -0.090198905 | 0.016929403 | -0.00142891 | -0.002460646 |
| p90 | -0.868722197 | -0.122809873 | -0.197934252 | -0.382486257 | -0.07136347 | 0.190528452 | 0.012931524 | -0.055799936 | 0.0064915 | 0.006995859 |
| ΔT1 time | 0.94629014 | 0.067719099 | 0.033539104 | 0.042550023 | 0.010878767 | 0.277033573 | -0.065494312 | 0.095293219 | -0.074087325 | 0.035896182 |
| K_trans_ | 0.964443119 | 0.004587516 | 0.009724224 | -0.024465449 | -0.168113575 | 0.095815472 | 0.105509625 | 0.070591948 | 0.11135213 | -0.056371323 |
| AUC | -0.769444701 | 0.074557364 | 0.540385467 | 0.063774724 | -0.306515679 | 0.043270045 | -0.095189615 | -0.028985391 | -0.020377615 | -0.013105809 |
| slope_max_ | -0.977490402 | -0.040922738 | 0.003695239 | -0.081731826 | 0.056469129 | -0.046947404 | 0.053276594 | 0.124447702 | -0.084062649 | -0.073206015 |
| T1 time | -0.975325609 | -0.020549903 | 0.103152228 | -0.050977571 | -0.02920064 | -0.074051244 | 0.02461426 | 0.132587714 | 0.06693832 | 0.077949956 |
| T2 time | -0.930264593 | 0.077185771 | 0.028088345 | 0.114648744 | 0.232625688 | 0.142542331 | -0.180558201 | 0.005276925 | 0.081010206 | -0.033046403 |

**Supplementary table 3.** Weights of the original variables on each calculated principal component (loadings) on day 6.

|  | **9d** | | | | | | | | | |
| --- | --- | --- | --- | --- | --- | --- | --- | --- | --- | --- |
| **Variable** | **PC1** | **PC2** | **PC3** | **PC4** | **PC5** | **PC6** | **PC7** | **PC8** | **PC9** | **PC10** |
| ADC_mean_ | 0.688989317 | -0.518020034 | 0.474263557 | -0.113980166 | 0.069101617 | 0.106303895 | 0.027022456 | -0.045659981 | -0.010704908 | -0.005135456 |
| kurtosis | 0.917719908 | -0.218159177 | -0.052732602 | -0.149847897 | 0.162147713 | -0.209093615 | -0.10931353 | -0.037529256 | 0.039804107 | 0.002709968 |
| range | -0.900179287 | -0.059536909 | -0.165140877 | -0.035691848 | 0.377027235 | 0.107102666 | -0.003016828 | 0.03716244 | 0.049553816 | 0.010997496 |
| p90 | 0.859474079 | -0.17788993 | -0.28135779 | -0.349703022 | -0.094376141 | 0.063810719 | 0.023121695 | 0.113556376 | -0.038994261 | 0.016618974 |
| ΔT1 time | -0.920079567 | 0.086803104 | -0.083659296 | -0.29336144 | -0.127715225 | 0.066677774 | -0.070579122 | -0.153904749 | 0.058554694 | 0.002271246 |
| K_trans_ | -0.936901446 | -0.100403875 | 0.251516698 | -0.064600964 | -0.104974017 | -0.0810055 | 0.052440595 | 0.096590626 | 0.117223907 | 0.036030051 |
| AUC | 0.534263314 | 0.757961853 | 0.282225144 | -0.215831901 | 0.112463242 | -0.005121287 | 0.022328288 | 0.016982207 | -0.01859526 | 0.003893609 |
| slope_max_ | 0.970045138 | 0.06635802 | -0.126755768 | 0.000245369 | -0.010665595 | 0.021468868 | 0.127292461 | -0.008349873 | 0.140779805 | -0.04330413 |
| T1 time | 0.97123985 | 0.05372629 | -0.111426523 | 0.127769085 | 0.019876539 | 0.019905567 | 0.095684421 | -0.104550086 | 0.013521179 | 0.063288788 |
| T2 time | 0.944538274 | 0.127799805 | 0.057028784 | 0.153534438 | -0.064383569 | 0.114603106 | -0.195899996 | 0.054223274 | 0.077191513 | 0.011616165 |

**Supplementary table 4.** Weights of the original variables on each calculated principal component (loadings) on day 9.
